# Pasta Drying Defects as a Novel Ingredient for Hard Dough Biscuits: Effect of Drying Temperature and Granulation on Its Functionality

**DOI:** 10.3390/foods13101487

**Published:** 2024-05-11

**Authors:** Jafar Mohammadzadeh Milani, Saeed Moammaei, Sepideh Haghighat Kharazi, Maryam Mohammadi Berenjestanaki

**Affiliations:** 1 Department of Food Science and Technology, Sari Agricultural Sciences and Natural Resources University, Sari P.O. Box 578, Iran; saeedmoammaei@gmail.com (S.M.); sepide_haghighat@yahoo.com (S.H.K.); mohammadibrnjst@yahoo.com (M.M.B.); 2 Department of Quality Control, Zarkam Company, Zar Industrial and Research Group, Hashtgerd 1991793963, Iran; 3 Department of Research and Development, Ardineh Iran Company Group, Ghaemshahr P.O. Box 47645/333, Iran

**Keywords:** pasta regrind, drying defects, drying temperature, regrinds granulation, by-product management

## Abstract

Various drying temperatures impact the texture of pasta and cause different drying defects. These by-products could reflect techno-functional characteristics which are suitable for cereal products. This research addresses the influence of low (LT) and high (HT) drying pasta defects with two granulations on the theoretical and functional characteristics of hard dough biscuits. By shifting from a LT to HT drying temperature, a higher onset and peak temperature was found due to the higher mobility of starch molecules with increasing crystalline stability. The lowest transition enthalpy of biscuit formulation was also observed for higher incorporation of fine HT pasta regrinds. The algebraic model of dough with consistography determined the poor-extensible gluten and a high resistance with a greater value of P/L and P indices for LT regrinds. Scanning electron microscopy revealed a heavy and dense texture with immersed starch granules for additional fine regrinds while coarse samples caused swell granules with greater diameter. Moreover, fine HT regrinds reflected the lowest L* value for biscuit due to heat gradient tension with the hard milling process which leads to protein denaturation with decreasing nitrogenous.

## 1. Introduction

Food wastes are generated in different forms and phases during physical or chemical processing. The aforementioned by-products are known as the functional source for recycling and conversion. One of the common processes to manage waste is using by-products in food ingredients which can retain the raw material nutrients [1]. Pasta and its products are prepared with semolina flour, water, and some optional ingredients. Blending and dampening with water can convert the flour into hydrated dough. To achieve the desired pasta texture, a continuous extrusion process must be applied. The last fundamental step involves the drying section, which comprises dehydrating final products to below 12% moisture [2]. There are some critical points of pasta production when considering the various sections and processes. Any technological maladjustment at each step causes defects in the final product. Pasta drying involves two critical characteristics: the drying temperature and dehydrating method. Due to the functional superiority of high-temperature dried pasta, the drying temperature and its periods have changed from 45- to 100 °C for some decades [3]. It could be concluded that the dried structure of pasta was considered a continuous matrix with entrapped starch granules in a coagulated region of the protein. By modifying the drying temperature, the pasta dehydrating treatment would be changed [3]. Generally, after supplying in the drying section, extruded dough moisture is removed from the surface via the hot air flow. Due to the applied gradient, moisture begins to diffuse from the core to the surface of the pasta strings [4]. Drying faults, such as internal cracking, burnt texture, and case hardening, occur individually at the beginning of the drying due to the application of high temperatures in a short time. In the pasta industry, by-products can be milled to the fine powder which is known as the pasta regrinds [5]. Based on the functionality of these regrinds, they can be blended with semolina to manufacture different types of pasta or mixed with flour to produce bread. These wastes are about 5 to 10 percent of the final dry products which are collected from different parts of production. To extend and optimize pasta regrinds, they could be applied as an ingredient in other starchy food matrices such as biscuits. They are long shelf-life products due to their low moisture content and are composed of wheat flour, sucrose, shortening, and other functional ingredients. Hard dough biscuits, in comparison with other wheat-based foods, need a weaker gluten-containing flour which is baked in flattened and thin layer forms. Moreover, pasta production causes starch damage and protein denaturation which leads to an enhanced harder and brittle structure. In this research, the effects of different levels of (LT and HT) pasta regrinds on dough rheology, as well as the microstructure and texture of biscuits and their intermolecular bonds, were investigated. By taking regrind granulations into consideration, their functionality as a coarse and fine additional ingredient was evaluated.

## 2. Materials and Method

### 2.1. Materials

Regarding the biscuit preparation, wheat flour was purchased from Gandomkoob flour mill company (Behshahr, Iran) with %10.5 protein content and %29 wet gluten. Glucose syrup (DE = 42, Zar Fructose Co., Hashtgerd, Iran), icing sugar, mineral salt (Taban Co., Tehran, Iran), sodium bicarbonate (Ardineh group, Ghaemshahr, Iran), sodium acid pyrophosphate (Fooding Co., Shanghai, China) and wheat starch (Zar Industrial Group, Hashtgerd, Iran) were bought commercially.

### 2.2. Pasta Defects Preparation

All of the low temperature dryers in pasta producing are the kind of cabinet dryers which use 50 to 65 °C with one or twin circulator fans. Low-temperature pasta defects were produced in the Maghoul pasta plant (Gorgan, Iran). The granular pasta flour was blended with B-carotene 10% (DSM, Heerlen, Switzerland) and hydrated to 30% moisture. A two-step parallel kneader with thin blades was employed to dampen the dough at 70 RPM. Then, a cylindrical extrusion powered using an 8.5 kW electromotor extruded the hydrated dough to prepare pasta with a length of 40 mm and an inner diameter of 15 mm. The extruded doughs were moved to the dryer and placed at the center of the chamber. To remove available water from the surface of the dough, twin air circulation fans were applied to create a great moisture gradient content. Due to the intensive thermal kinetics of keeping the temperature at 65 °C [6], surface burnings occurred at the beginning of drying. Burnt-texture spaghetti was milled in two steps and sieved with a laboratory plansifter; samples were categorized into fine and coarse granulations. High-temperature dried pasta was purchased from Zarmacaron Company (Hashtgerd, Iran) and dried at 95 °C in a continuous spaghetti line. After preparation, samples were milled and sieved as mentioned earlier.

### 2.3. Biscuit Preparation

According to [7], optimized formulations with some modifications were used to prepare the biscuit. Aimed at 100 g of biscuit, 53 g wheat flour, 13 g icing sugar, 12 g margarine, 0.4 g sodium bicarbonate, 0.4 g sodium acid pyrophosphate, 0.2 g distilled monoglycerides, 1 g wheat starch, 1.8 g glucose syrup, and 0.2 g salt were selected as the main recipe to prepare the dough. By adding 18 mL water, all ingredients were mixed using a mixer (KitchenAid Inc., St. Joseph, MI, USA) at level VI for 7 min. After mixing, they were hand kneaded for 30 s and rested for 15 min at 4 °C. The dough pieces were then sheeted (Sinmag-SM-520S, Kuala Lumpur, Malaysia) by diminishing their thickness to 2 mm and rested for another 15 min. Finally, the doughs were shaped with a cutting ring of 25 mm and 2 cm diameter. The biscuits were then baked in the air electrical oven at 175 °C and airflow set (level I) for 12 min (UNOX- Italy- oven section). The prepared biscuits were cooled for 60 min and stored in zip bags for further tests. Additional samples were prepared by substituting wheat flour with 30, 50, and 60 percent pasta defect flours (regrind) which were dried at low and high temperatures with coarse and fine granulations.

### 2.4. Physicochemical Characteristics of Flour

The analysis of biscuit flour with LT and HT regrinds for physicochemical characteristics was conducted for the moisture, ash content, protein, and wet gluten using NIRs instruments (Foss, DS2500 L, Hillerød, Denmark). By adding 50 g of each treatment, the sample cup was loaded onto the device. Different wavelength ranges with collected spectral dates would predict various quality attributes [8].

### 2.5. Alveographic Assay

Dough tenacity (P), dough extensibility (L), index of swelling (G), dough baking strength (W), and configuration of the curve (P/L) of wheat flour incorporated with pasta regrinds were calculated at the optimum hydration using Chopin NG Alveograph^®^ (Villeneuve-la-Garenne, France) [9].

### 2.6. Consistography Assay

Blended wheat flour with various mass ratios of pasta defect flours was analyzed using a consistograph (Chopin Technologies, Villeneuve-la-Garenne, France) to calculate the water absorption capacity. 

### 2.7. Physical Characteristics of Biscuit

#### 2.7.1. Dimensions and Spread Ratio

The 2D dimensions of each biscuit were measured with a Vernier Calliper (CD-6″ CSX, Mitutoyo Corporation, Sakado, Japan). The diameter was calculated on two orthogonal sections which was provided as the average value. By measuring the height of well-formed biscuits in two rows and their horizontal measurements, the spread ratio was obtained by dividing diameter by height. The measurements were applied to five biscuit samples [10].

#### 2.7.2. Three-Point Bend Test

To measure the hardness value of baked biscuits, a 3-point bending rig (HDP/3 PB) with a 10 g load cell (Brookfield^®^ CT3, Engineering Labs, Middleboro, MA, USA) was conducted. Samples were placed between the support beams at a distance of 1 mm. A central beam was adjusted to move and break the biscuit. Pre-test, test, and post-test speeds were set, respectively, at 1.0, 2.0, and 10.0 [7]. All the measurements were taken in triplicate.

#### 2.7.3. Crust Color 

The color of the baked biscuits with different formulations was measured in terms of Hunter L, a, and b values using a spectro-colorimeter [11]. All the tests were completed in triplicate.

### 2.8. Fourier Transform Infrared Spectroscopy Analysis (FTIR)

FTIR spectroscopy of the biscuit incorporated with pasta drying defects was analyzed using a FTIR spectrophotometer (FT-IR, Cary 630, Santa Clara, Agilent, CA, USA). To study the FTIR spectra, 0.5 mg of each sample was mounted on the device and scanned from 600 cm^−1^ to 4000 cm^−1^ [12].

### 2.9. Solvent Retention Capacity

The solvent retention capacity method was applied to evaluate the functional variables as follows: water holding capacity, swelling power of glutenin, and starch damage as described by [13]. Samples were mixed with respective solvents (distilled water, 5% Na_2_CO_3_, 5% lactic acid). Then, the suspension was sent to a centrifuge device and the test was performed at 1000× *g* for 15 min. After drip drying, the swollen section was measured. All the measurements were taken in triplicate.

### 2.10. Differential Scanning Calorimetry (DSC)

Differential scanning calorimetry (Zufa, 11054, Shanghai, China) was performed to measure the starch gelatinization. A total of 3–6 mg of biscuit flour with pasta regrinds was weighed and hydrated with a distilled water ratio of 1/3. The applied temperature gradient with 10 c/min varied from 30 °C to 95 °C [14].

### 2.11. Scanning Electron Microscopy (SEM)

To determine the effect of low- and high-temperature pasta defects on biscuit textures, the microstructures of samples were analyzed through SEM (SNE-4500M, Suwon, Korea). The freeze-dried biscuit samples were mounted on an aluminum sample holder, having a diameter of (12 mm) and covered with a thin layer of gold. The magnifications of 30× and 150× were selected under an acceleration voltage of 20 kV to photograph the microstructure [15].

### 2.12. Statistical Analysis

A one-way ANOVA of the Infostat v.9.0 software was used to evaluate significant differences (*p* ≤ 0.05) among samples for some experiments. Differences between means were identified using Duncan’s multiple comparison test. The treatment analysis was performed in three replicates.

## 3. Results and Discussion

### 3.1. Mixed Flour Characteristics

The compositional results of wheat flour containing pasta regrind are presented in Table 1. Each regrind substitution reflects different moisture content for the aforementioned treatments which were in line with the pasta drying temperature (LT and HT) and milling pressure (Fine and Coarse). Particle size distribution could influence the water content and hydration rate of each treatment. Fine regrinds indicate the lowest moisture content with the highest water absorption among the samples. On the other hand, wet gluten and protein content was raised with the incorporation of coarse pasta regrinds.

### 3.2. Alveographic Properties

Rheological analysis of biscuit flour with LT and HT regrinds was performed based on the determinations using the Chopin Alveolab to determine various phases of dough processing during the baking process (sheeting, rounding, and molding). The results presented significant differences (*p* ≤ 0.05) for most of the alveographic parameters (Table 2). An optimal P/L factor could be 0.2 for short dough products, whereas the ratio nearing 1.0 was chosen for voluminous bread. B5HC5 and B4HF6 had a P/L ranging between 0.19 and 0.2 like control flour [16]. However, biscuit dough incorporated with low-temperature pasta regrinds showed the highest P/L, whereas the extensibility parameter of dough which was reflected by L indices decreased drastically. These values showed poor-extensible gluten structure with high resistance to deformation [17]. To the best of our knowledge, these results could be related to the lower protein denaturation of LT regrinds due to the low-temperature drying and extruding during pasta making. Based on the close relation between W indices and gluten structure, biscuit dough samples could be categorized based on the W value. B4HF6 and B5HC5 indicated the lowest values for W indices. This finding revealed that high-temperature drying regrinds with fine granulation caused poor gluten strength for the control sample. Additionally, the P indices of dough samples indicated the same tendency of P/L value. It was also related positively to the water-holding capacity and negatively to moisture content. B4LF6 presented the lowest value of P indices due to higher starch content of fine regrinds for low-temperature drying methods [18].

### 3.3. Physical Properties

**Figure 1:**
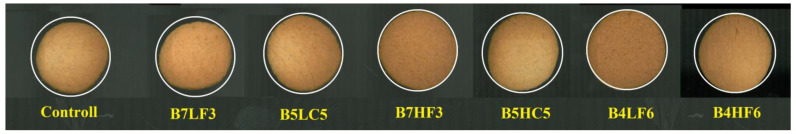
Representative image of formulated biscuits slices and their geometrical differences. Control = 100% biscuit flour; B7LF3 = 70% biscuit flour incorporated with 30% fine LT regrind; B4LF6 = 40% biscuit flour incorporated with 60% fine LT regrind; B5LC5 = 50% biscuit flour incorporated with 50% coarse LT regrind; B7HF3 = 70% biscuit flour incorporated with 30% fine HT regrind; B4HF6 = 40% biscuit flour incorporated with 60% fine HT regrind; B5HC5 = 50% biscuit flour incorporated with 50% coarse HT regrind.

The physical characteristics of the biscuits, including thickness, diameter, weight, spread ratio, and hardness are listed in Table 3. The spread ratio of cooked biscuits was increased with an increase in coarse LT and HT pasta regrinds. This parameter is influenced by the protein structure, which affects the binding properties of biscuit texture. Moreover, the used flour granulation could be related to the water-holding capacity of the flour. As water-holding capacity increased, spread ratio parameters showed a higher value [9]. B5LC5 and B5HC5, with the incorporation of coarse regrinds, reflected the highest spread ratio among the treatments. Formulated biscuit diameters varied from 32.60 to 34.50 mm, whereas the thickness changed from 3.00 to 5.40 mm (Figure 1). To the best of our knowledge, these results could be due to the addition of LT and HT fine regrinds which influence the biscuit texture by increasing diameter and decreasing thickness [19].

In the three-point bending test, the hardness parameter of biscuits was measured under the title of texture resistance to the breaking. The results indicated that the least hardness was related to the B5HC5 and B5LC5, which were formulated by the addition of coarse LT and HT regrinds. This result could be understood as there being more porosity structure with weakened gluten network prepared using granular regrinds [20].

### 3.4. Color

The crust color of baked biscuits was analyzed using L (lightness), a (redness), and b (yellowness) parameters (Table 3). Colorimetry measurements indicated that formulated biscuits with HT and fine regrinds had a lower L* value in comparison with the other samples. They also gave significantly higher a* and b* degrees in the analysis which reflect a significantly high red hue. The differences in color parameters were especially found for B5LC5 with higher intensity of Maillard browning reaction [6]. This finding could be related to the drying and milling processes of pasta. Coarse regrinds with low-temperature drying imposed a lower mechanical force and heat tension. The aforementioned parameters caused lower protein denaturation, and more amino acids with reducing sugars would be available for the Maillard browning reaction. Conversely, a high drying temperature with a hard milling process for fine granulation leads to protein denaturation with decreasing nitrogenous bases, thereby reducing the protein percentage [21].

### 3.5. FTIR

**Figure 2:**
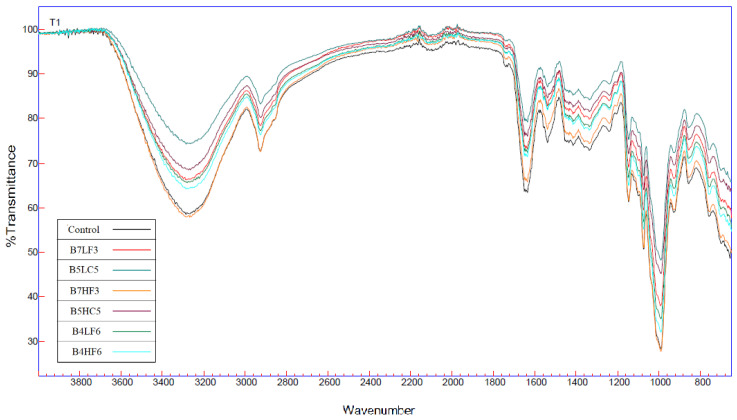
Fourier transform infrared spectroscopy of control and formulated biscuits. Control = 100% biscuit flour; B7LF3 = 70% biscuit flour incorporated with 30% fine LT regrind; B4LF6 = 40% biscuit flour incorporated with 60% fine LT regrind; B5LC5 = 50% biscuit flour incorporated with 50% coarse LT regrind; B7HF3 = 70% biscuit flour incorporated with 30% fine HT regrind; B4HF6 = 40% biscuit flour incorporated with 60% fine HT regrind; B5HC5 = 50% biscuit flour incorporated with 50% coarse HT regrind.

FTIR spectroscopy was performed to detect functional groups of pasta regrinds with biscuit flour (Figure 2). Infrared spectroscopy analysis proved that all biscuit flour with the incorporation of LT and HT regrinds had close spectra peaks in the same wavenumber regions. Common peaks at 994.01 cm^−1^ to 994.4 cm^−1^ presented starchy content due to the vibration of glycosidic bonds [22]. The transmittance bands at 1076.81 cm^−1^ to 1077.16 cm^−1^ regions also displayed the stretching vibration of the C–O–H group and reflected the amorphous region of starch. B7HF3 showed the lowest transmittance percentage due to the presence of fine regrinds, which leads to a more amorphous state of starch. The region of frequencies around 3272.9 and 3277.03 cm^−1^ were attributed mainly to O-H bond starching [11]. By increasing the coarse form of pasta regrinds, the intensity of O-H bonds were also reinforced. This result might be related to intra- and intermolecular hydrogen bonds of water which were involved with starch. Additionally, important peaks in the region of 1637.17 cm^−1^ to 1646.96 cm^−1^ specified the presence of the amide I band which originated from the stretching vibration of the C=O bond. Higher absorptions were recorded for control and B7HF3 samples in comparison with the other treatments. This result indicated that high-temperature drying pasta regrinds with fine granulation had no significant side effect for the gluten networks of the control flour. However, the lowest intensity of amid I band for B5LC5 was observed as a result of coarse low-temperature pasta regrinds supplementation.

### 3.6. Solvent Retention Capacity

The solvent retention of biscuit flours with the incorporation of HT and LT pasta regrinds were influenced by the main components such as protein and starch (Table 3). Each component solvation in the selected solvent determines the swelling behavior of polymers. Distilled water (W-RC), lactic acid in water (La-RC), and sodium carbonate (Na_2_CO_3_-RC) were used for water retention capacity, analyzing the swelling power of glutenin and starch damage. By decreasing the swelling power of glutenin, a reduction in La-RC value for B4LF6 was observed. Moreover, the drying process with multiple grinding actions for B7LF3 caused a higher water-holding capacity (W-RC), due to more starch damage and the gelatinization degree. The water retention capacity of B7LF3 increased significantly with the addition of fine and low-temperature drying regrinds in comparison with the control sample [23]. The value of La-RC for fine HT regrinds was greater than Na_2_CO_3_-RC. This outcome could be related to the outstanding role of glutenin for water binding against the LT and biscuit flour. The value of Na_2_CO_3_-RC indicates an increasing trend for formulations with HT regrinds. B5HC5, with the highest sodium carbonate swelling power value, reflects the starch damage in the retention capacity. In addition to the naturally damaged starch content through the action of α-amylases, regrinds could present gelatinized and retrograded granules with greater damaged starch content. A high-temperature drying process with the milling step could be known as the main factor damaging starch in regrinds.

### 3.7. DSC

The gelatinization of starch with changes in DSC endotherms was analyzed via differential scanning calorimetry measurements (Table 3). The results indicated the effect of drying processes (LT or HT) on the thermal behavior of starch granules in biscuits. Increasing the drying temperature of pasta regrinds resulted in a higher onset and peak temperature in comparison with the control sample. This result in B4FH6 sample could be backed by the higher mobility of starch molecules with increasing crystalline stability according to the results of [24]. The lowest transition enthalpy rate was also found for B4FH6, with the highest incorporation of high-temperature drying pasta regrind. This result could be expected by imposing an extrusion process and higher drying temperature which create gelatinized and retrograded starch granules. The aforementioned composition also required less energy to melt granules which is in agreement with the previous study [25]. On the other hand, for LT pasta regrinds, starch granules were initiated to melt with a 5 to 10 °C difference lower than the control sample. The reason for the results could be due to the lower temperature requirement for melting starch in fine-LT regrinds, which were incorporated with biscuit flour resulting in a lower stability product during heating.

### 3.8. Microstructural Characteristics

**Figure 3:**
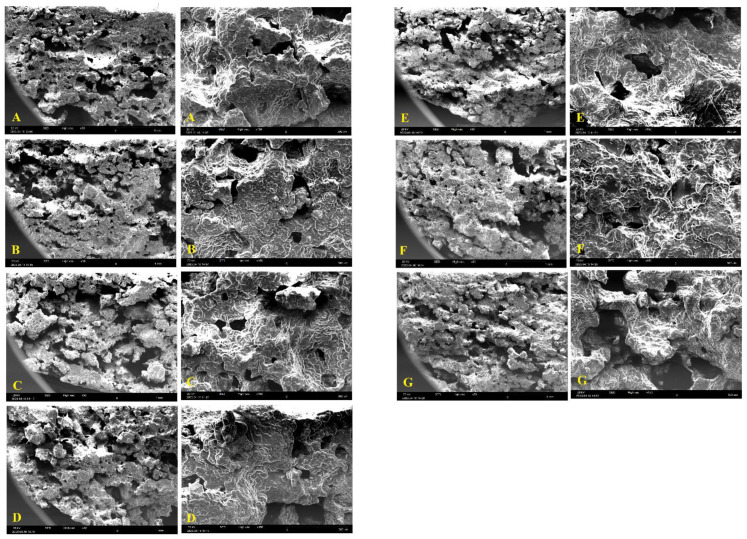
Scanning electron microscopy (SEM) of biscuit microstructure for (**A**) Control, (**B**) B7LF3, (**C**) B5LC5, (**D**) B7HF3, (**E**) B5HC5, (**F**) B4LF6, and (**G**) B4HF6 (Magnifications = 35× and 100×). Control = 100% biscuit flour; B7LF3 = 70% biscuit flour incorporated with 30% fine LT regrind; B4LF6 = 40% biscuit flour incorporated with 60% fine LT regrind; B5LC5 = 50% biscuit flour incorporated with 50% coarse LT regrind; B7HF3 = 70% biscuit flour incorporated with 30% fine HT regrind; B4HF6 = 40% biscuit flour incorporated with 60% fine HT regrind; B5HC5 = 50% biscuit flour incorporated with 50% coarse HT regrind.

The microstructures of baked biscuits were analyzed through SEM to observe the characteristics of the immersed starch granules. Visual magnifications of 30× and 150× of all biscuit formulations were depicted in (Figure 3). SEM micrographs indicated the smooth starch granules for the control biscuit. For the B5LC5 sample, coarse LT pasta regrinds exhibited more starch granules with greater diameter in some regions. Swollen starch granules of the intended biscuit reflect the higher capacity of water holding due to the lower extrusion pressure, heat tension, and grinding force in the coarse LT method. However, the fine granulation of additional pasta regrinds caused the immersed starch granules with greater dispersion [26]. The B4LF6 sample microstructure with fine LT regrinds revealed a heavy and dense texture with a high number of fused granules. By observation of biscuit samples with fine HT regrinds, starch granules lost their granular identity proportional to HT regrinds added. Imposing a higher extrusion and grinding pressure to starch granules exhibited greater starch damage values for fine HT and LT regrinds. This reason for the finding could be backed by the unbalanced ability of water absorption which exposed the granules to more aggressive enzymes [26]. These results were in agreement with the alveographic parameter (P/L) variation for rheological analysis.

## 4. Conclusions

The experiment results highlighted that LT and HT pasta regrinds with various granulations (*p* ≤ 0.05) influence the rheological behavior of biscuit doughs and the final product. The results showed that as much as the HT regrinds increased, the influence of starch damage and gelatinization degree increased and a greater capacity of water-holding (W-RC) was observed. Moreover, the reduction in extensibility for biscuit dough incorporated with LT regrinds was determined by increasing P/L indices. It was also concluded that the P parameter presents the same tendency of P/L for treatments. B5LC5 and B5HC5, with the incorporation of coarse regrind, reflected the highest spread ratio with more starch granules and greater diameter in some regions in SEM observation; whereas, fine granulation pasta regrinds caused the immersed starch granules with greater dispersion. Calculated 3-point-bend analysis of biscuits indicated that coarse LT and HT regrinds reflected the least content of hardness which could be related to more porosity structure with weakened gluten network. This research indicated that pasta regrinds, based on their techno-functional characteristics of drying temperature and granulation, could be feasible ingredients for products that do not require long-proofing such as biscuits. 

## Figures and Tables

**Table 1:** Biscuit formulation with compositional characteristics of NIRs analysis.

Sample	Ingredient (%)					Moisture	Ash	Protein	Wet Gluten	Water Absorption
	Biscuit Flour	Fine-Low Temperature Pasta Regrind	Coarse-Low Temperature Pasta Regrind	Fine-High Temperature Pasta Regrind	Coarse-High Temperature Pasta Regrind					
Control	100	-	-	-	-	13.50	0.5	11.73	27.96	53.93
B7LF3	70	30	-	-	-	12.47	0.53	11.62	27.37	55.21
B4LF6	40	60	-	-	-	11.68	0.56	11.49	26.98	56.18
B5LC5	50	-	50	-	-	12.26	0.51	11.51	28.12	56.09
B7HF3	70	-	-	30	-	12.70	0.52	11.60	27.23	55.67
B4HF6	40	-	-	60	-	11.89	0.54	11.41	26.61	57.21
B5HC5	50	-	-	-	50	12.42	0.51	11.54	28.30	55.55

Control = 100% biscuit flour; B7LF3 = 70% biscuit flour incorporated with 30% fine LT regrind; B4LF6 = 40% biscuit flour incorporated with 60% fine LT regrind; B5LC5 = 50% biscuit flour incorporated with 50% coarse LT regrind; B7HF3 = 70% biscuit flour incorporated with 30% fine HT regrind; B4HF6 = 40% biscuit flour incorporated with 60% fine HT regrind; B5HC5 = 50% biscuit flour incorporated with 50% coarse HT regrind.

**Table 2:** Alveographic measurements of control and substituted doughs.

Parameters	Control	B7LF3	B4LF6	B5LC5	B7HF3	B4HF6	B5HC5
P	58.66 ± 2.51 ^d^	114.33 ± 114.33 ^bc^	180 ± 37.04 ^a^	144.33 ± 5.51 ^bc^	171.66 ± 3.05 ^ab^	23.66 ± 19.35 ^e^	28 ± 16.09 ^e^
G	27.2 ± 1.73 ^a^	16.33 ± 0.33 ^b^	12.63 ± 2.54 ^b^	11.03 ± 0.23 ^b^	11.06 ± 0.45 ^b^	25.53 ± 6.68 ^a^	25.53 ± 5.51 ^a^
W	271 ± 12.49 ^a^	241.33 ± 241.3 ^ab^	226.33 ± 74.57 ^ab^	164 ± 3 ^b^	198.66 ± 16.56 ^ab^	66 ± 65 ^c^	72.33 ± 68.41 ^c^
P/L	0.39 ± 0.058 ^d^	2.11 ± 2.11 ^c^	5.73 ± 1.33 ^b^	5.86 ± 0.46 ^b^	6.89 ± 0.48 ^a^	0.2 ± 0.05 ^d^	0.197 ± 0.05 ^d^
L	151.33 ± 16.74 ^a^	54 ± 1.73 ^bc^	33.33 ± 12.42 ^c^	24.66 ± 1.15 ^c^	25 ± 2 ^c^	109 ± 65.39 ^ab^	136.33 ± 53.57 ^a^
Hydration	54.8±0.099^a^	52.2±0.099^b^	48±0.099^f^	49.2±0.099^d^	50.9±0.099^c^	48.6±0.099^e^	48±0.099^f^

Control = 100% biscuit flour; B7LF3 = 70% biscuit flour incorporated with 30% fine LT regrind; B4LF6 = 40% biscuit flour incorporated with 60% fine LT regrind; B5LC5 = 50% biscuit flour incorporated with 50% coarse LT regrind; B7HF3 = 70% biscuit flour incorporated with 30% fine HT regrind; B4HF6 = 40% biscuit flour incorporated with 60% fine HT regrind; B5HC5 = 50% biscuit flour incorporated with 50% coarse HT regrind. P-maximum pressure (mm H_2_O), L-extensibility (mm), G-swelling index (mm), W-energy (J 10^-4^). P/L- ratio. Means (n = 3) with the different superscript lowercase letters within a column are significantly different (*p* < 0.05).

**Table 3:** Dimensional and physico-chemical characteristics, thermal transition parameters and SRC retention capacity for baked biscuits.

Test	Parameters	Control	B7LF3	B4LF6	B5LC5	B7HF3	B4HF6	B5HC5
Physical properties	Diameter	32.68 ± 1.17 ^c^	32.616 ± 0.076 ^c^	33.78 ± 0.58 ^abc^	33.183 ± 0.17 ^bc^	33.63 ± 0.63 ^abc^	34.316 ± 0.8 ^ab^	34.5 ± 0.390512 ^a^
	Thickness	5.28 ± 0.30 ^a^	3.733 ± 0.25 ^bc^	3.5 ± 0.1 ^bc^	3.066 ± 0.42 ^c^	3.5 ± 0.18 ^bc^	3.25 ± 0.13 ^bc^	3.416 ± 0.26 ^bc^
	Weight	12.04 ± 0.09 ^a^	10.19 ± 0.08 ^d^	11.013 ± 0.08 ^b^	8.933 ± 0.057 ^e^	10.59 ± 0.056 ^c^	12.21 ± 0.02 ^a^	12.03 ± 0.1 ^a^
	Spread ratio	0.69 ± 0.04 ^c^	0.91 ± 0.005774 ^b^	0.88 ± 0.01 ^b^	1.2 ± 0.025166 ^a^	1.026 ± 0.011547 ^a^	1.03 ± 0.005 ^a^	1.0 ± 0.01 ^a^
	Hardness (g)	1251.83 ± 95.24 ^a^	1171.33 ± 192.87 ^a^	1195.166 ± 440.01 ^a^	413.16 ± 46.40 ^c^	898.33 ± 89.31 ^ab^	716.5 ± 54.43 ^bc^	548.5 ± 57.17 ^bc^
Color	L	71.06 ± 1.52 ^a^	66.54 ± 7.22 ^ab^	69.78 ± 4.22 ^a^	51.48 ± 2.46 ^c^	60.86 ± 5.87 ^b^	64.70 ± 4.71 ^ab^	47.92 ± 2.48 ^c^
	a	6.92 ± 0.64 ^b^	8.85 ± 4.84 ^ab^	1.27 ± 3.7 ^c^	13.03 ± 1.7 ^a^	9.42 ± 1.25 ^ab^	7.11 ± 2.42 ^b^	12.62 ± 2.38 ^a^
	b	36.32 ± 0.21 ^a^	34.53 ± 3.75 ^ab^	30.4 ± 1.44 ^c^	26.57 ± 0.92 ^d^	30.22 ± 2.55 ^c^	31.44 ± 1.89 ^bc^	25 ± 1 ^d^
SRC	Water	3.14 ± 0.02 ^ab^	3.23 ± 0.34 ^a^	2.86 ± 0.037 ^cd^	3.019 ± 0.01 ^abc^	2.95 ± 0.01 ^bcd^	2.75 ± 0.04 ^d^	3.07 ± 0.02 ^abc^
	Lactic acid 5% (*w*/*w*)	2.51 ± 0.11 ^b^	2.34 ± 0.13 ^c^	2.06 ± 0.06 ^d^	2.83 ± 0.015 ^a^	2.45 ± 0.05 ^bc^	2.73 ± 0.011 ^a^	2.73 ± 0.011 ^a^
	Sodium carbonate 5% (*w*/*w*)	2.96 ± 0.05 ^a^	2.60 ± 0.11 ^b^	2.53 ± 0.11 ^b^	2.31 ± 0.09 ^c^	2.82 ± 0.01 ^a^	2.56 ± 0.13 ^b^	2.95 ± 0.005 ^a^
DSC	T_o_ (°C)	71.6	60.1	64.4	65.7	66.3	77	71.5
	T_p_ (°C)	87.2	56.3	87	88.1	91.9	92.3	90.4
	T_c_ (°C)	95.1	95.5	95.7	98.1	98.3	96.5	98.2
	Transition enthalpy rate	65.32%	74.95	76.43%	69.92	69.60%	0.09%	21.82%

Control = 100% biscuit flour; B7LF3 = 70% biscuit flour incorporated with 30% fine LT regrind; B4LF6 = 40% biscuit flour incorporated with 60% fine LT regrind; B5LC5 = 50% biscuit flour incorporated with 50% coarse LT regrind; B7HF3 = 70% biscuit flour incorporated with 30% fine HT regrind; B4HF6 = 40% biscuit flour incorporated with 60% fine HT regrind; B5HC5 = 50% biscuit flour incorporated with 50% coarse HT regrind. Data were expressed as the mean of three replicates, given with their standard deviation. Means with the different superscript lowercase letters within a column are significantly different (*p* < 0.05).

## Data Availability

The original contributions presented in the study are included in the article, further inquiries can be directed to the corresponding author.
